# *Cytinus hypocistis* (L.) L. subsp. *macranthus Wettst*.: Nutritional Characterization

**DOI:** 10.3390/molecules24061111

**Published:** 2019-03-20

**Authors:** Ana Rita Silva, Ângela Fernandes, Pablo A. García, Lillian Barros, Isabel C.F.R. Ferreira

**Affiliations:** 1 Centro de Investigação de Montanha (CIMO), Instituto Politécnico de Bragança, Campus de Santa Apolónia, 5300-253 Bragança, Portugal; silva-ana@ipb.pt (A.R.S.); afeitor@ipb.pt (A.F.); lillian@ipb.pt (L.B.); 2 Departamento de Ciencias Farmacéuticas, Facultad de Farmacia, CIETUS-IBSAL, University of Salamanca, Campus Miguel de Unamuno, 37007 Salamanca, Spain; pabloagg@usal.es

**Keywords:** *Cytinus hypocistis* (L.) L. subsp. *macranthus* Wettst., famine food, holoparasite, nectar, nutritional and chemical characterization, wild edible plant

## Abstract

The habit of eating wild plants in Europe is often associated with times of famine; an example of such is the nectar of *Cytinus hypocistis* (L.) L., a parasitic plant. To the authors’ best knowledge, there are no studies on its nutritional and chemical composition; thus, the whole *C. hypocistis* (L.) L. subsp. *macranthus* Wettst. plant (CH) and its nectar (NCH) were nutritionally and chemically characterized. The proximate composition of CH and NCH were very similar in terms of energy, ash, and carbohydrate content. Protein and fat were approximately 2-fold higher in NCH, and crude fiber was 4.6-fold higher in CH compared to NCH. Fructose, glucose, sucrose, and trehalose were the free sugars present in both samples. Oxalic, malic, and citric acids were the identified organic acids in both samples, with citric acid as the most abundant molecule. For both samples, polyunsaturated and saturated fatty acids (PUFA and SFA, respectively) predominate over monounsaturated fatty acids (MUFA) due to the significant contribution of linoleic and palmitic acids, respectively. However, unsaturated fatty acids (UFA) prevail over SFA in CH and NCH. Therefore, CH proved to be an excellent source of nutritional compounds, which supports its use during past periods of scarcity.

## 1. Introduction

Wild edible plants have been an integral part of human nutrition since ancient times, and many species now considered as weeds were food substitutes, the most common individual subsistence strategy in times of want and starvation for numerous cultures [1,2,3]. Indeed, all the early studies on the use of wild food plants in Europe, beginning in the 19th century to approximately the 1960s, captured the history of famine and the use of wild plants as a means of basic survival [2]. Despite agricultural societies’ primary dependence on crop plants, the tradition of eating wild plants has not completely disappeared [4,5,6,7]. An example of such is the parasitic plant *Cytinus hypocistis* [7,8,9]. Approximately 1% of angiosperms are parasitic [10,11,12], and one of the most extreme manifestations of this type of parasitism is found within the Cytinaceae family, composed of the *Cytinus*, *Bdallophyton*, and *Bdallophytum* genera [8,13]. *Cytinus* are rootless, stemless, and leafless holoparasites with a vegetative body reduced to an endophytic system that grows exclusively inside its host root, and from which nutrients and water are absorbed [13]. This genus occurs in the Mediterranean region, South Africa, and Madagascar, and the flowers are only visible when they emerge from the host tissue during the reproductive period [8]. In Europe, there are two recognized species of *Cytinus*—*Cytinus hypocistis* (L.) L. and *Cytinus ruber* (Fourr.) ex Fritsch—that parasitize roots of *Cistus* and *Halimium* spp. and two genera of shrub plants within the *Cistaceae* family. *Cytinus hypocistis* (L.) L. is divided into four subspecies, each with a distinct host range: subsp. *hypocistis* parasitize on various *Cistus* and *Halimium* spp., subsp. mac*ranthus* parasitize on *Halimium* spp., subsp. *orientalis* parasitize on *Cistus parviflorus*, and subsp. *pityusensis* parasitize on *Cistus clusii* [6,14,15,16].

Three different studies on wild plants traditionally used for human consumption in Portugal and Spain quoted *C. hypocistis* as famine food; its nectar was sucked as sweets or spread on rye bread during the working day to avoid hunger pains [6,7,16]. From the nutritional point of view, flowers can be divided into three major components (pollen, nectar, and petals) and other parts, which can play a role in human nutrition [17]. Nectar is the second most important component; it is usually a sweetish liquid which contains a balanced mixture of sugars (fructose, glucose, and sucrose), amino acids, proteins, inorganic ions, lipids, organic acids, phenolic substances, alkaloids, and terpenoids, among others [18].

According to a semi-quantitative approach that compares the cultural importance of 97 wild edible plant species of the Iberian Peninsula, *C. hypocistis* occupies position 44 in the ranking [7]. Despite the cultural relevance of this plant, its chemical characterization is largely unknown [8,19,20], and to the author’s best knowledge, its nutritional composition is not yet identified. For a comprehensive discussion to help bridge this gap, and since *C. hypocistis* nectar accounts for 70 ± 0.5% of its flower weight, the authors compared the obtained data with published results from other studies on different edible flowers. Therefore, *C. hypocistis* subsp. *macranthus* Wettst. was nutritionally characterized based on its protein, fat, ash, fiber, and carbohydrate content, following which we calculated its energetic value. Furthermore, its individual content in sugars, organic acids, and fatty acids was also determined.

## 2. Results and Discussion

According to the literature, water is the main constituent (70 to 95%) and carbohydrates are the most abundant macronutrient (42.4 to 90.2 g/100 g dry weight basis—dw) in edible flowers [21]. The nutritional profiles of the whole *C. hypocistis* (L.) L. subsp. *macranthus* Wettst. plant (CH) and its nectar (NCH) are presented in Table 1 and were within the range for edible flowers reported in the literature [21]. The humidity contents of CH and NCH were 78% and 25%, respectively. Protein (9.4 versus 4.90 g/100 g dw) and fat (1.4 versus 0.67 g/100 g dw) values were approximately 2-fold higher in NCH in comparison to CH. Unlike ash (2.87 g/100 g dw for CH and 3.05 g/100 g dw for NCH) and carbohydrates (86.8 g/100 g dw for CH and 85.1 g/100 g dw for NCH), where the content in both samples were very similar, crude fiber was 4.6-fold higher in CH compared to NCH (4.76 versus 1.03 g/100 g dw). Altogether, these factors contributed to a very similar energetic value for both samples (382.4 kcal/100 g dw for CH and 392.9 kcal g/100 g dw for NCH).

The soluble sugar composition of the two samples is also shown in Table 1. Two reducing (i.e., fructose and glucose) and two non-reducing (i.e., sucrose and trehalose) sugars were detected in both samples. Fructose is known to be the sweetest of all naturally occurring carbohydrates [22] and was the main sugar present in CH, almost 9-fold higher (6.3 g/100 g dw) than in NCH (0.71 g/100 g dw). Glucose was also almost 9-fold higher in CH (1.92 g/100 g dw) than in NCH (0.22 g/100 g dw). Although sucrose was the main soluble sugar present in NCH, its concentration was almost 2-fold lower (0.85 g/100 g dw) than in CH (1.37 g/100 g dw). Contrary to the other three sugars, trehalose content was similar for both samples, 0.95 g/100 g dw in CH and 0.80 g/100 g dw in NCH. The total sugar content was 4-fold higher in the whole plant (10.5 g/100 g dw versus 2.58 g/100 g dw), mainly due to the contribution of fructose. Nectar is described in the literature as containing a balanced mixture of sugars [17], and NCH was found to have a very similar content of fructose, sucrose, and trehalose, confirming this information. 

**Figure 1:**
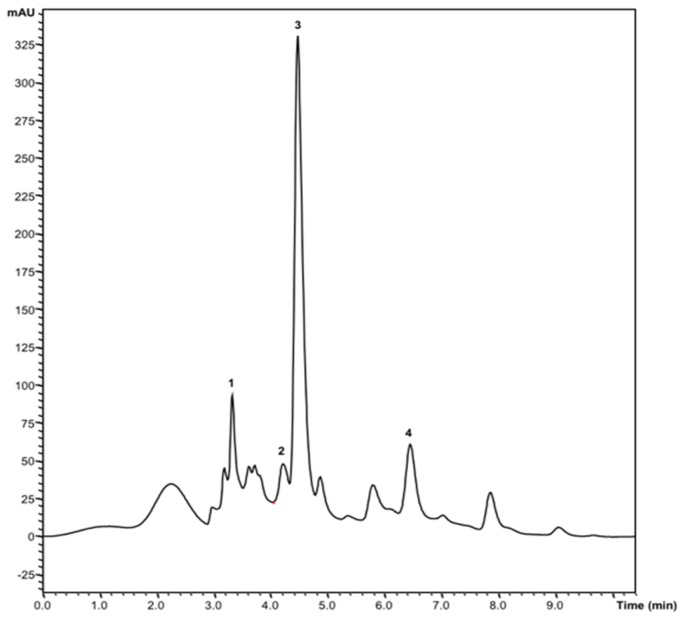
Organic acids chromatogram profile of the CH sample at 215 nm: 1—oxalic acid, 2—malic acid, 3—ascorbic acid, and 4—citric acid.

Three different organic acids were identified in both samples (CH and NCH): oxalic (0.030 g/100 g dw versus traces), malic (0.40 g/100 g dw versus 0.45 g/100 g dw), and citric acids (0.41 g/100 g dw versus 1.48 g/100 g dw). Contrarily, ascorbic acid was only detected in NCH (0.180 g/100 g dw) and traces of shikimic acid were detected in CH. As presented in Figure 1, the total organic acids content was 2.48-fold higher in NCH (2.11 g/100 g dw) compared to CH (0.85 g/100 g dw). All detected organic acids are of the utmost importance for human metabolism since they are described as beneficial for a healthy diet [23]. 

Regarding tocopherols content, only traces of α-tocopherol isoform were detected in CH.

Results regarding the fatty acids composition of CH and NCH are given in Table 2. The fatty acids profile showed 25 compounds for CH and 26 for NCH. Polyunsaturated fatty acids (PUFA) were the major group, followed by saturated fatty acids (SFA) and monounsaturated fatty acids (MUFA). Humans lack the enzymes required to produce the two essential fatty acids: ω-3 PUFA-α-linolenic and ω-6 PUFA-linoleic. Although the synthesis rate may not be sufficient to meet human requirements, and it is, hence, recommended that good sources of these fatty acids are also included in the diet, humans can elongate dietary α-linolenic acid to the long chain ω-3 PUFAs, namely eicosapentaenoic and docosahexaenoic acids [24]. PUFA corresponds to 46.95% of the fatty acids present in CH and 49% in NCH, mainly due to the high content of linoleic acid in both samples (40.08% and 39.903%, respectively). Linoleic and α-linolenic acids are present in high percentages in some edible flowers (>50%), such as *Calendula officinalis* L. and *Trifolium angustifolium* L. [25]. CH (42.14%) and NCH (43.62%) stayed just below the 50% cut-off line. Linoleic and α-linolenic acids have important roles in human growth and development, as well as in the prevention and treatment of coronary artery diseases, hypertension, diabetes, arthritis, other inflammatory and autoimmune disorders, and cancer [25,26,27,28,29,30]. SFA is the second group of fatty acids with similar predominance in CH (35.56%) and NCH (35.36%), largely due to the high content of palmitic acid (24.12 and 24.76%, respectively). Palmitic acid is one of the most common SFA found in edible plants. Although it is associated with increased risk of developing cardiovascular diseases [31], oxidative DNA damage, DNA strand breakage, necrosis, and apoptosis in human cells in vitro [32,33], when consumed with other fatty acids, like PUFAs, SFA are unlikely to have any significant impact on human health [25,32,34]. A recent review highlighted that further research is needed to unveil the true advantages and disadvantages induced by palmitic acid consumption [35]. CH and NCH also contain other saturated fatty acids in lower concentrations, such as stearic (CH: 5.19%, NCH: 4.79%), arachidic (CH: 1.87%, NCH: 1.453%), and behenic acids (CH: 1.86%, NCH: 1.57%). MUFA makes up the smallest contribution to the fatty acids content in CH (17.5%) and NCH (15.31%), mainly due to the presence of oleic acid (CH: 15.4%, NCH: 13.70%). Both samples presented small percentages of palmitoleic (CH: 0.662%, NCH: 0.628%), elaidic (CH: 1.10%, NCH: 0.861%), and eicosanoic acids (CH: 0.366%, NCH: 0.121%). As it has been shown, olive oil induces its hypotensive effects through the action of oleic acid and, according to Fernandes et al. [25], one of the highest percentages of this fatty acid present in edible flowers was found in *Gundelia tournefortii* L. buds (28.5%) [25,36]. For both samples (Table 2), PUFA and SFA predominate over MUFA due to the significant contribution of linoleic and palmitic acids, respectively. However, unsaturated fatty acids (UFA) prevail over SFA (64.44% versus 35.56% in CH and 6.9% versus 35.36% in NCH). According to the literature, with the exception observed in calendula flowers (23.3%), unsaturated fatty acids predominate over saturated ones for edible flowers, usually being higher than 53% [25]. According to Fernandes et al. [25], in general all edible flowers studied until now showed high ratios (above 0.45) of PUFA/SFA, which are known to help reduce the risk of cardiovascular diseases [34], and the *Cytinus hypocistis* (L.) L. plant is no exception. The PUFA/SFA ratios for CH and NCH were 1.320 and 1.37, respectively. 

## 3. Materials and Methods 

### 3.1. Reagents and Standards

Acetonitrile (99.9%), *n*-hexane (95%), and ethyl acetate (99.8%) were of HPLC grade from Fisher Scientific (Lisbon, Portugal). All the individual compounds were of HPLC or GC grade, the fatty acids methyl ester (FAME) reference standard mixture 37 (standard 47,885-U) was purchased from Sigma (St. Louis, MO, USA), as well as other individual fatty acid isomers, l-ascorbic acid, tocopherols (α-, β-, γ-, and δ-isoforms), and sugars (d(–)-fructose, d(+)-glucose anhydrous, d(+)-melezitose hydrate, d(+)-sucrose, and d(+)-trehalose). All other chemicals and solvents were of analytical grade purity and purchased from common suppliers. Water was treated in a Milli-Q water purification system (TGI Pure Water Systems, Greenville, SC, USA).

### 3.2. Plant Material

**Figure 2:**
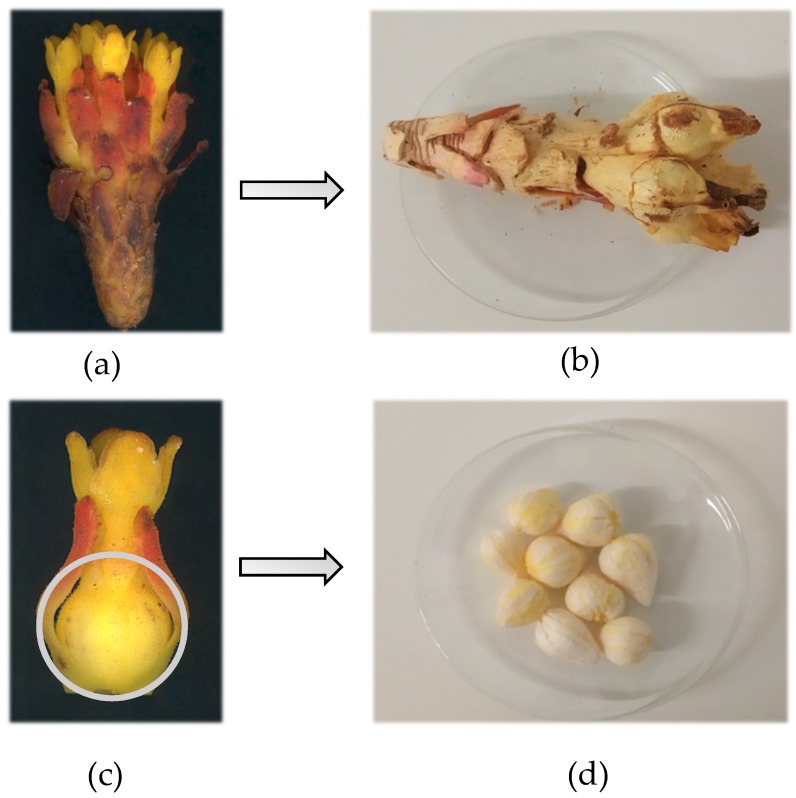
*Cytinus hypocistis* subsp. *macranthus* Wettst. (L.) L.: (**a**) fresh plant, (**b**) lyophilized plant, (**c**) fresh flower, and (**d**) lyophilized nectar.

*Cytinus hypocistis* (L.) L. subsp. *macranthus* Wettst plants were collected in July 2018 from the host species *Halimium lasianthum* subsp. *alyssoides* (Lam.) Greuter at three different locations in Castro Daire, Portugal. Plant identification and characterization were conducted using Flora Europaea [14] botanical criteria and the online platform flora.on coordinated by the Portuguese Botanical Association. The fresh material was thoroughly cleaned with deionized water to remove all soil, drained on absorbent tissue, and frozen at −30 °C. After lyophilization (FreeZone 4.5 model 7750031, Labconco, KS, USA), as shown in Figure 2, dried plants were separated into two different samples, whole plant (CH) and nectar (NCH), and reduced to a fine dried powder (20 mesh). The dried powders were stored at −30 °C and protected from light until further analysis. 

### 3.3. Nutritional Value of Cytinus hypocistis (L.) L. subsp. macranthus Wettst

The proximate composition (i.e., proteins, fat, ash, fiber, and carbohydrates) and energetic value were evaluated in CH and NCH. The crude protein content of the samples was determined following the macro-Kjeldahl method [N × 6.25, AOAC (Official Methods of Analysis of AOAC INTERNATIONAL) 991.02], the total fat using a Soxhlet apparatus with petroleum ether as the extraction solvent (AOAC 989.05), and the ash content by sample incineration at 550 ± 15 °C (AOAC 935.42) [37]. Fiber was determined based on the solubilization of non-cellulosic compounds using sulfuric acid and potassium hydroxide solutions (FIWE Fiber Analyzers). Total available carbohydrates were calculated by its difference, using the following equation: Total carbohydrates (g/100 g) = 100 − (g fat + g protein + g ash + g fiber). Total energy was calculated according to the following equation: Energy (kcal/100 g) = 4 × (g proteins + g carbohydrates) + 9 × (g fat) + 2 × (g fiber).

### 3.4. Chemical Characterization of Cytinus hypocistis (L.) L. subsp. macranthus Wettst

#### 3.4.1. Soluble Sugars

To determine the composition of the soluble sugars, 1 g of each sample (CH and NCH) was mixed with melezitose (internal standard—IS, 25 mg/mL) and extracted with 40 mL of 80% aqueous ethanol at 80 °C, followed by solvent evaporation and fat removal with consecutive ethyl ether washes as previously described by Pereira et al. [38,39]. High-performance liquid chromatography (Knauer, Smartline system 1000, Berlin, Germany) coupled to a refractive index detector (HPLC-RI) was the chosen methodology and the data were analyzed using Clarity 2.4 Software (DataApex, Prague, Czech Republic). HPLC consisted of integrated equipment with a pump (Knauer, Smartline system 1000, Berlin, Germany), degasser (Smartline manager 5000), auto-sampler (AS-2057 Jasco, Easton, MD, USA), and an RI detector (Knauer Smartline 2300). Data were analyzed using Clarity 2.4 Software (DataApex). The chromatographic separation was achieved with a Eurospher 100-5 NH_2_ column (4.6 × 250 mm, 5 µm, Knauer) operating at 30 °C (7971 R Graceoven). The mobile phase was acetonitrile/deionized water (70:30, *v*/*v*) at a flow rate of 1 mL/min. Identification was carried out by comparing authentic standard retention times, while quantification was achieved using the IS method (DataApex, Podohradska, Czech Republic), with calibration curves constructed from authentic standards. Soluble sugars were further expressed in g per 100 g of dry weight (dw). 

#### 3.4.2. Organic Acids

Metaphosphoric acid (4.5%) was added to 1 g of the sample; the mixture was then protected from light and incubated (with agitation) for 20 min at room temperature. After sample filtration, organic acids were determined using a Shimadzu 20A series UFLC (Shimadzu Corporation, Kyoto, Japan) coupled to photodiode array detector (PDA) [39]. Separation was achieved on a SphereClone (Phenomenex, Torrance, CA, USA) reverse phase C_18_ column (5 μm, 250 mm × 4.6 mm i.d—internal diameter.) thermostatted at 35 °C. The elution was performed with sulphuric acid (3.6 mM) using a flow rate of 0.8 mL/min. Detection was carried out in a PDA using 215 and 245 nm (for ascorbic acid) as preferred wavelengths. For the quantitative analysis, calibration curves with known concentrations of commercial standards were constructed, and the organic acids present in the two samples were determined by peak area comparison at 215 nm and 245 nm (for ascorbic acid). The results were expressed in g per 100 g dw.

#### 3.4.3. Fatty Acids

Fatty acid content was investigated after trans-esterification of the lipid fraction obtained through Soxhlet extraction as previously described by Pinela et al. [38]. The samples were filtered with a 0.2 µm nylon filter (Whatman) and analyzed by gas–liquid chromatography (DANI 1000, Contone, Switzerland) with flame ionization detection (GC-FID)/capillary column. The analysis was carried out with a split/splitless injector, an FID at 260 °C, and a Zebron-Kame column (30 m × 0.25 mm i.d. × 0.20 µm film thickness, Phenomenex, Torrance, CA, USA). The oven temperature program was as follows: The initial temperature of the column was 100 °C, held for 2 min, then a 10 °C/min ramp to 140 °C, 3 °C/min ramp to 190 °C, 30 °C/min ramp to 260 °C, held for 2 min. The carrier gas (hydrogen) flow rate was 1.1 mL/min, measured at 100 °C. Split injection (1:50) was carried out at 250 °C. Fatty acid identification and quantification were achieved by comparing the relative retention times of the fatty acids methyl ester peaks with standards. The results were recorded and processed using CSW 1.7 software (DataApex 1.7) and expressed in the relative percentage for each fatty acid.

#### 3.4.4. Tocopherols

Hexane solutions of butyl-hydroxy-toluene (10 mg/mL; 100 μL) and tocol (internal standard, 400 μL at 50 μg/mL) were added to 500 mg of the sample prior to extraction, as formerly described by Pinela et al. [38]. The combination was then homogenized with 4 mL of methanol by vortex mixing (1 min), followed by 4 mL of hexane (by vortex mixing for 1 min). After sample homogenization, a saturated NaCl aqueous solution (2 mL) was added, the mixture was combined (vortex mixed for 1 min), centrifuged (5 min, 4000 *g*), and the clear upper layer carefully transferred to a vial. Sample extraction with hexane was performed three times. The combined extracts (i.e., the clear layer) were dried under a nitrogen stream, dissolved in 2 mL of n-hexane, dehydrated with anhydrous sodium sulphate, filtered through a 0.2 µm nylon filter (Whatman), transferred into a dark injection vial, and analyzed by HPLC (Knauer, Smartline system 1000, Berlin, Germany) coupled to a fluorescence detector (FP-2020; Jasco, Easton, MD, USA) [38]. The chromatographic separation was achieved with a Polyamide II (250 mm × 4.6 mm i.d.) normal-phase column from YMC Waters operating at 30 °C. The mobile phase used was a mixture of n-hexane and ethyl acetate (70:30, *v/v*) at a flow rate of 1 mL/min, and the injection volume was 20 µL. The fluorescence detector was programmed for excitation at 290 nm and emission at 330 nm. The compounds were identified by chromatographic comparisons with authentic standards. Quantification was based on calibration curves obtained from commercial standards of each compound using the IS methodology. The results were expressed in µg/100 g dw.

### 3.5. Statistical Analysis

CH and NCH samples were used for all the assays carried out in triplicate and the results were expressed as mean values and standard deviations (SD). The results were analyzed using a Student’s *t*-test, in order to determine the significant difference between two different samples with a 5% significance level (IBM SPSS Statistics, version 22.0. SPSS, Armonk, NY, USA).

## 4. Conclusions

*Cytinus hypocistis* subsp. *macranthus* Wettst. (L.) L. nectar has proven to be a good and balanced source of sugars and other carbohydrates, ash, proteins, lipids, and organic acids. This was a novel study of the nutritional and chemical characterization of this parasitic edible plant and allowed for a better understanding of the reasons behind the use of this plant in the past as a source of nutritional compounds during famine periods. Further investigation is needed to clarify potential applications of *C. hypocistis*. Therefore, a phytochemical characterization of the most bioactive molecules, such as phenolic compounds, could be determined and correlated to its biological properties in order to understand attributes given to this plant species. 

## Figures and Tables

**Table 1:** The nutritional value, soluble sugars, and organic acids composition of *Cytinus hypocistis* subsp. *macranthus* Wettst. (L.) L. using the whole plant (CH) and its nectar (NCH). Mean ± SD.

Moisture (%)	CH	NCH	Student’s *t*-Test *p*-Value
	78 ± 1	25 ± 1	<0.001
**Nutritional value**	**g/100 g dw**		
Fat	0.67 ± 0.03	1.4 ± 0.1	<0.001
Proteins	4.90 ± 0.07	9.4 ± 0.3	<0.001
Ash	2.87 ± 0.02	3.05 ± 0.05	0.005
Fiber	4.8 ± 0.1	1.03 ± 0.05	<0.001
Carbohydrates	86.8 ± 0.2	85.1 ± 0.4	0.002
Energy contribution (kcal/100 g dw)	382.4 ± 0.1	392.9 ± 0.1	<0.001
**Soluble sugars**	**g/100 g dw**		
Fructose	6.3 ± 0.1	0.71 ± 0.01	<0.001
Glucose	1.92 ± 0.05	0.22 ± 0.02	<0.001
Sucrose	1.37 ± 0.05	0.85 ± 0.01	<0.001
Trehalose	0.95 ± 0.02	0.80 ± 0.04	0.001
Total	10.5 ± 0.2	2.58 ± 0.07	<0.001
**Organic acids**	**g/100 g dw**		
Oxalic acid	0.030 ± 0.001	tr.	-
Malic acid	0.40 ± 0.01	0.45 ± 0.02	0.007
Shikimic acid	tr.	nd.	-
Ascorbic acid	nd.	0.180 ± 0.002	-
Citric acid	0.41 ± 0.01	1.48 ± 0.01	<0.001
Total	0.85 ± 0.02	2.11 ± 0.03	<0.001

dw—dry weight basis, tr.—traces, and nd.—not detected.

**Table 2:** Fatty acids composition of *Cytinus hypocistis* subsp. *macranthus* Wettst. (L.) L. using CH and NCH (Mean ± SD).

Fatty Acids (Relative Percentage, %)	CH	NCH	Student’s *t*-Test *p*-Value
Caproic acid (C6:0)	nd.	0.100 ± 0.001	-
Caprilic acid (C8:0)	0.030 ± 0.003	0.033 ± 0.001	0.178
Capric acid (C10:0)	0.037 ± 0.003	0.036 ± 0.001	0.011
Undecylic acid (C11:0)	0.016 ± 0.001	0.042 ± 0.001	<0.001
Lauric acid (C12:0)	0.315 ± 0.002	0.268± 0.001	<0.001
Myristic acid (C14:0)	0.425 ± 0.001	0.384 ± 0.001	<0.001
Pentadecylic acid (C15:0)	0.15 ± 0.01	0.13 ± 0.01	0.001
Palmitic acid (C16:0)	24.12 ± 0.07	24.76 ± 0.02	<0.001
Palmitoleic acid (C16:1)	0.662 ± 0.001	0.628± 0.001	<0.001
Margaric acid (C17:0)	0.311 ± 0.004	0.305 ± 0.001	<0.001
Stearic acid (C18:0)	5.19 ± 0.04	4.79 ± 0.01	<0.001
Elaidic acid (C18:1n9t)	1.10 ± 0.02	0.86 ± 0.01	<0.001
Oleic acid (C18:1n9c)	15.4 ± 0.1	13.7 ± 0.1	<0.001
Linolelaidic acid (C18:2n6t)	2.16 ± 0.01	1.88 ± 0.01	0.001
Linoleic acid (C18:2n6c)	40.08 ± 0.02	39.90 ± 0.03	<0.001
γ-Linolenic acid (C18:3n6)	1.088 ± 0.001	0.940 ± 0.005	<0.001
α-Linolenic acid (C18:3n3)	2.07 ± 0.06	3.72 ± 0.02	<0.001
Arachidic acid (C20:0)	1.87 ± 0.01	1.45 ± 0.01	<0.001
Eicosanoic acid (C20:1)	0.366 ± 0.004	0.121 ± 0.004	<0.001
cis-11,14-Eicosadienoic acid (C20:2)	1.471 ± 0.005	1.273 ± 0.001	0.001
Heneicosanoic acid (C21:0)	0.22 ± 0.01	0.25 ± 0.01	0.001
Arachidonic acid (C20:4n6)	0.028 ± 0.001	0.034 ± 0.002	<0.001
Behenic acid (C22:0)	1.86 ± 0.06	1.57 ± 0.01	0.001
cis-13,16-Docosadienoic acid (C22:2)	0.058 ± 0.001	0.037 ± 0.001	<0.001
Tricosanoic acid (C23:0)	0.182 ± 0.003	0.191 ± 0.004	0.003
Lignoceric acid (C24:0)	0.83 ± 0.03	2.60 ± 0.02	<0.001
SFA	35.56 ± 0.09	35.36 ± 0.02	0.006
MUFA	17.5 ± 0.1	15.3 ± 0.1	<0.001
PUFA	46.95 ± 0.04	49 ± 1	0.022
UFA	64.4 ± 0.1	63.8 ± 0.8	0.282
PUFA/SFA	1.32 ± 0.01	1.37 ± 0.02	0.015

dw—dry weight basis, nd.—not detected, SFA—saturated fatty acids, MUFA—monounsaturated fatty acids, PUFA—polyunsaturated fatty acids, and UFA—unsaturated fatty acids.

## References

[1] Torija-Isasa M.E., Matallana-González M.C. (2016). A Historical Perspective of Wild Plant Foods in the Mediterranean Area. Mediterranean Wild Edible Plants.

[2] Łuczaj Ł., Pieroni A., Tardío J., Pardo-de-Santayana M., Sõukand R., Svanberg I., Kalle R. (2012). Wild food plant use in 21st century Europe: The disappearance of old traditions and the search for new cuisines involving wild edibles. Acta Soc. Bot. Pol..

[3] Carvalho A.M., Barata A.M. (2016). The Consumption of Wild Edible Plants. Wild Plants, Mushrooms and Nuts.

[4] Jman Redzic S. (2006). Wild Edible Plants and Their Traditional Use in the Human Nutrition in Bosnia-Herzegovina. Ecol. Food Nutr..

[5] Nebel S., Pieroni A., Heinrich M. (2006). Ta chòrta: Wild edible greens used in the Graecanic area in Calabria, Southern Italy. Appetite.

[6] Tarío J., Pardo-de-Santayana M., Morales R. (2006). Ethnobotanical review of wild edible plants in Spain. Bot. J. Linn. Soc..

[7] Pardo-de-Santayana M., Tardío J., Blanco E., Carvalho A., Lastra J., San Miguel E., Morales R. (2007). Traditional knowledge of wild edible plants used in the northwest of the Iberian Peninsula (Spain and Portugal): A comparative study. J. Ethnobiol. Ethnomed..

[8] Zucca P., Pintus M., Manzo G., Nieddu M., Steri D., Rinaldi A.C. (2015). Antimicrobial, antioxidant and anti-tyrosinase properties of extracts of the Mediterranean parasitic plant Cytinus hypocistis. BMC Res. Notes.

[9] Quivik F.L. (2011). The Illusory Boundary: Environment and Technology in History.

[10] Rubiales D., Heide-Jørgensen H.S. (2011). Parasitic Plants. Encycl. Life Sci..

[11] Těšitel J. (2016). Functional biology of parasitic plants: A review. Plant Ecol. Evol..

[12] Westwood J.H., Yoder J.I., Timko M.P., dePamphilis C.W. (2010). The evolution of parasitism in plants. Trends Plant Sci..

[13] De Vega C., Ortiz P.L., Arista M., Talavera S. (2007). The endophytic system of mediterranean Cytinus (cytinaceae) developing on five host Cistaceae species. Ann. Bot..

[14] Tutin T.G., Burges N.A., Chater A.O., Edmondson J.R., Heywood V.H., Moore D.M., Valentine D.H., Walters S.M., Webb D.A. (1993). Flora Europaea.

[15] Thorogood C.J., Hiscock S.J. (2007). Host Specificity in the Parasitic Plant *Cytinus hypocistis*. Res. Lett. Ecol..

[16] Carvalho A.M., Morales R., Pardo de Santayana M., Pieroni A., Puri R. (2010). Persistence of Wild Food and Wild Medicinal Plant Knowledge in a Northeastern Region of Portugal. Ethnobotany in the New Era: People, Health and Wild Plant Resources.

[17] Mlcek J., Rop O. (2011). Fresh edible flowers of ornamental plants—A new source of nutraceutical foods. Trends Food Sci. Technol..

[18] Nicolson S.W., Nepi M., Pacini E. (2007). Nectaries and Nectar.

[19] Schildknecht H., Herb R., Kunzelmann P. (1985). Die Chemie der Schmarotzerblumen, II. Isoterchebin: Struktur des gelben Ellagitannin-Farbstoffes ausCytinus hypocistis (Rafflesiaceae). Liebigs Ann. der Chemie.

[20] Magiatis P., Pratsinis H., Kalpoutzakis E., Konstantinidou A., Davaris P., Skaltsounis A.-L. (2001). Hydrolyzable Tannins, the Active Constituents of Three Greek Cytinus Taxa against Several Tumor Cell Lines. Biol. Pharm. Bull..

[21] Fernandes L., Casal S., Pereira J.A., Saraiva J.A., Ramalhosa E. (2017). Edible flowers: A review of the nutritional, antioxidant, antimicrobial properties and effects on human health. J. Food Compos. Anal..

[22] Hanover L.M., White J.S. (1993). Manufacturing, composition, and applications of fructose. Am. J. Clin Nutr..

[23] Zhou Y., He W., Zheng W., Tan Q., Xie Z., Zheng C., Hu C. (2018). Fruit sugar and organic acid were significantly related to fruit Mg of six citrus cultivars. Food Chem..

[24] Lunn J., Theobald H.E. (2006). The health effects of dietary unsaturated fatty acids. Nutr. Bull..

[25] Fernandes L., Ramalhosa E., Pereira J.A., Saraiva J.A., Casal S. (2018). The Unexplored Potential of Edible Flowers Lipids. Agriculture.

[26] Boden G., Sargrad K., Homko C., Mozzoli M., Stein T.P. (2005). Effect of a Low-Carbohydrate Diet on Appetite, Blood Glucose Levels, and Insulin Resistance in Obese Patients with Type 2 Diabetes. Ann. Intern. Med..

[27] Peyron-Caso E., Taverna M., Guerre-Millo M., Véronèse A., Pacher N., Slama G., Rizkalla S.W. (2002). Dietary (n-3) Polyunsaturated Fatty Acids Up-Regulate Plasma Leptin in Insulin-Resistant Rats. J. Nutr..

[28] Simopoulos A.P. (1999). Essential fatty acids in health and chronic disease. Am. J. Clin. Nutr..

[29] Mozaffarian D., Geelen A., Brouwer I.A., Geleijnse J.M., Zock P.L., Katan M.B. (2005). Effect of Fish Oil on Heart Rate in Humans. Circulation.

[30] Tortosa-Caparrós E., Navas-Carrillo D., Marín F., Orenes-Piñero E. (2017). Anti-inflammatory effects of omega 3 and omega 6 polyunsaturated fatty acids in cardiovascular disease and metabolic syndrome. Crit. Rev. Food Sci. Nutr..

[31] Ness A. (2004). Diet, Nutrition and the Prevention of Chronic Diseases. WHO Technical Report Series 916. Report of a Joint WHO/FSA Expert Consultation. Int. J. Epidemiol..

[32] Ricchi M., Odoardi M.R., Carulli L., Anzivino C., Ballestri S., Pinetti A., Fantoni L.I., Marra F., Bertolotti M., Banni S. (2009). Differential effect of oleic and palmitic acid on lipid accumulation and apoptosis in cultured hepatocytes. J. Gastroenterol. Hepatol..

[33] Carvalho I.S., Teixeira M.C., Brodelius M. (2011). Fatty acids profile of selected Artemisia spp. plants: Health promotion. LWT - Food Sci. Technol..

[34] French M.A., Sundram K., Clandinin M.T. (2002). Cholesterolaemic effect of palmitic acid in relation to other dietary fatty acids. Asia Pac. J. Clin. Nutr..

[35] Mancini A., Imperlini E., Nigro E., Montagnese C., Daniele A., Orrù S., Buono P. (2015). Biological and Nutritional Properties of Palm Oil and Palmitic Acid: Effects on Health. Molecules.

[36] Terés S., Barceló-Coblijn G., Benet M., Alvarez R., Bressani R., Halver J.E., Escribá P.V. (2008). Oleic acid content is responsible for the reduction in blood pressure induced by olive oil. Proc. Natl. Acad. Sci. U S A.

[37] Latimer G.W. (2016). AOAC Official Methods of Analysis of AOAC INTERNATIONAL.

[38] Pinela J., Barros L., Carvalho A.M., Ferreira I.C.F.R. (2011). Influence of the drying method in the antioxidant potential and chemical composition of four shrubby flowering plants from the tribe Genisteae (Fabaceae). Food Chem. Toxicol..

[39] Pereira E., Barros L., Calhelha R.C., Dueñas M., Carvalho A.M., Santos-Buelga C., Ferreira I.C. (2014). Bioactivity and phytochemical characterization of *Arenaria montana* L.. Food Funct..

